# Supplementary material for: TERT Promoter Methylation Is Oxygen-Sensitive and Regulates Telomerase Activity
Source: Biomolecules. 2024 Jan 19;14(1):131. doi: 10.3390/biom14010131 (PMC10813121; doi:10.3390/biom14010131)

Supplementary Figure S1. Cell viability assessed with WST1 assay in air oxygen and physoxia conditions. A) SHEF1, B) SHEF2 in air oxygen (21% O_2) and physoxia (2% O_2 and WKS) and images represent cell morphology of SHEF1 **(A)** and SHEF2 **(B)** cells. * indicates $p<0.05$ versus air oxygen.

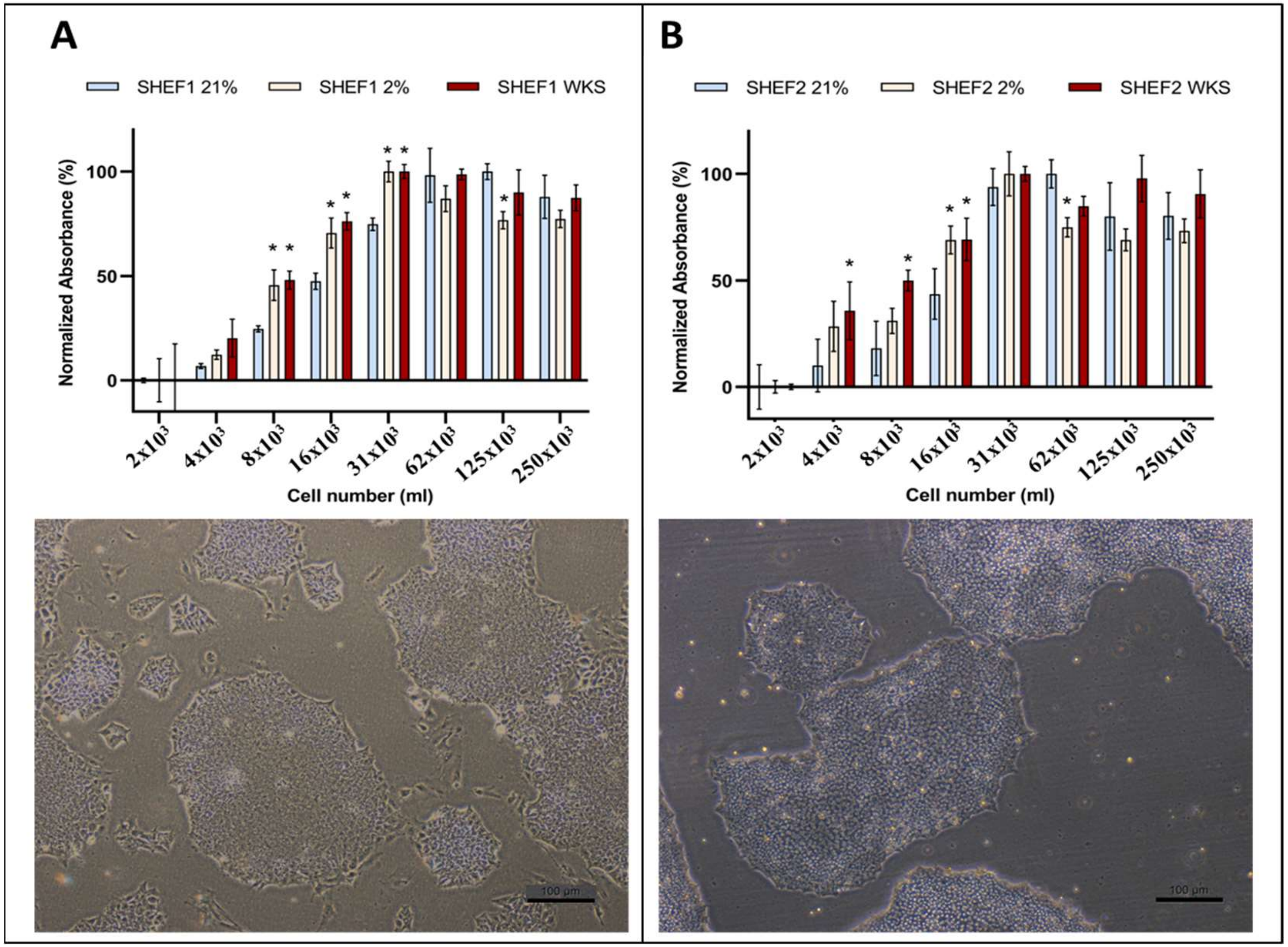

Supplementary Figure S2. Loss of pluripotency marker expression during differentiation of hESC. The expression of pluripotency markers (Oct-3/4, Nanog, SSEA-1, SSEA-4, and alkaline phosphatase) were analyzed in undifferentiated and differentiated ESCs using immunofluorescence staining. (A) SHEF1, (B) SHEF2 cells.

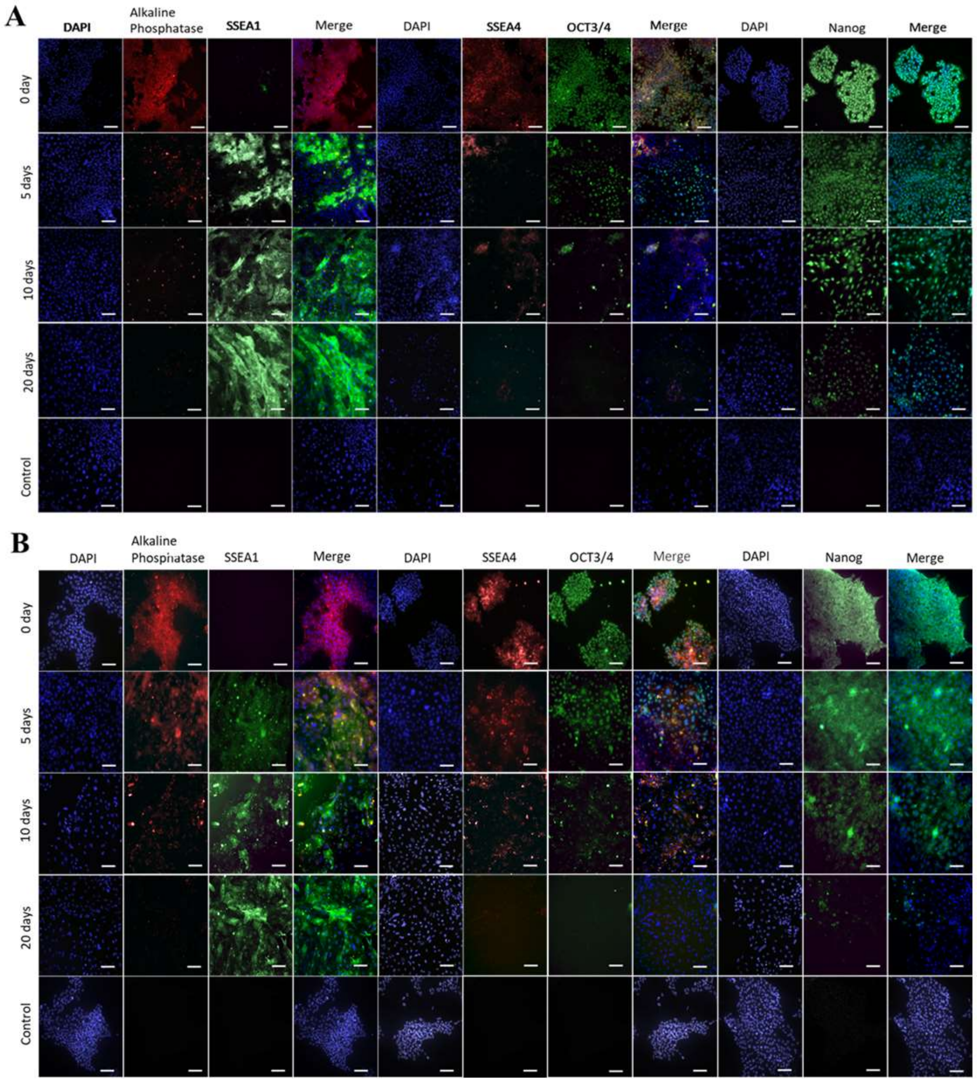

Supplementary Figure S3. Increased mesodermal markers expression in monolayer differentiated hESCs. Expression of three germ layer (Ectoderm (NES), Mesoderm (KDR, TBXT), and Endoderm (AFP)) differentiation markers was analyzed in SHEF1 (A) and SHEF2 (B) in 21% AO, 2% PG and 2% WKS. The y-axis indicates expression (-ΔΔCT) is normalized to GAPDH. Data are represented as n = 3x3 (three biological samples and each sample has three replicates), *p<0.05, **p<0.01, ***p<0.001 vs air oxygen. D indicates day of differentiation.

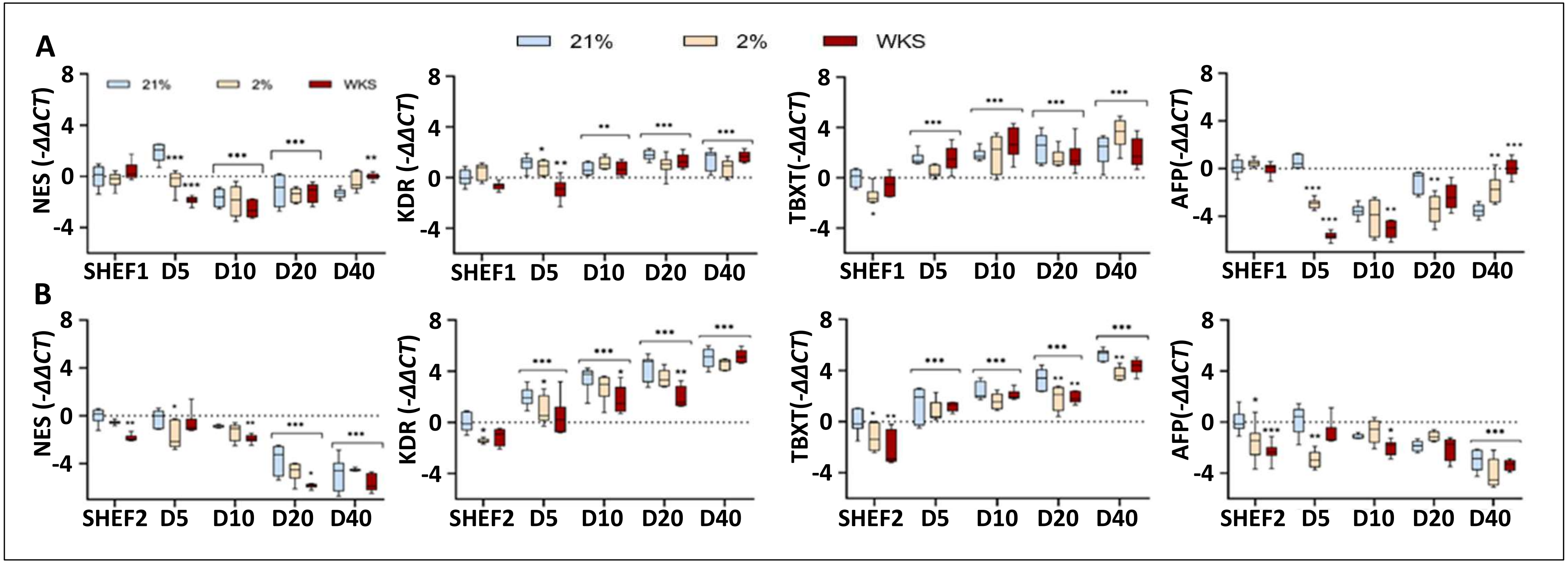

Supplementary Figure S4. Physoxia slows downregulation of TERT, telomerase activity, and rate of telomere shortening in differentiating SHEF1 (A) and SHEF2 (B) cultured in air oxygen (21% AO) and physiological oxygen conditions (2% PG and 2% WKS) are presented for TERT expression, telomerase activity, and telomere shortening across 40 days differentiation. Data are presented as mean ± standard deviation (SD), n = 3X3, *p<0.05, **p<0.01, ***p<0.001. x-axis; D indicates day of differentiation.

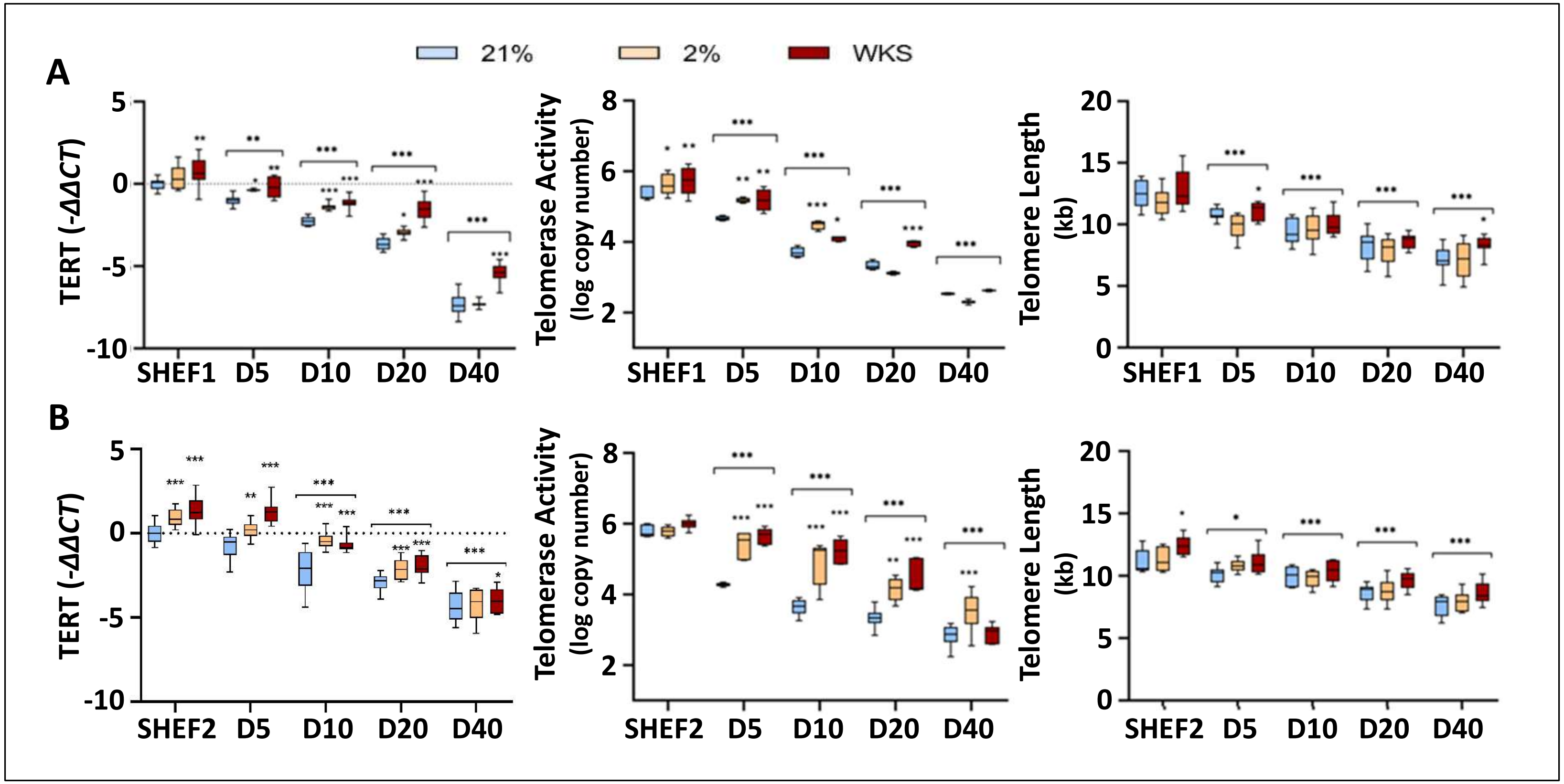

Supplementary Figure S5: TERT protein expression in SHEF1 (A) and SHEF2 (B) cells

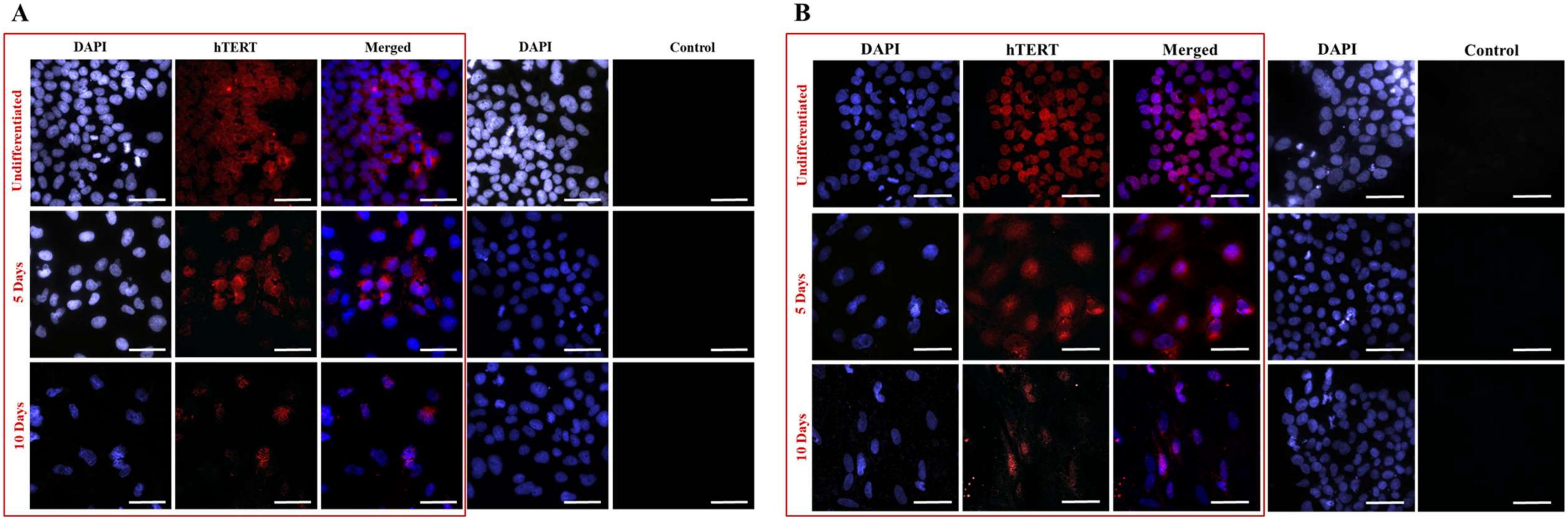

Supplementary Figure S6. Differentiated SHEF1 and SHEF2 cells population doubling time. SHEF1 (A) and SHEF2 (B) cells differentiated in three oxygen conditions. The X-axis represents days in cell culture, and Y-axis shows the population doublings during 40 days of differentiation. The results are illustrated with three independent samples (n=3) for each condition. Data are represented as mean ± standard deviation (SD), *p<0.05.

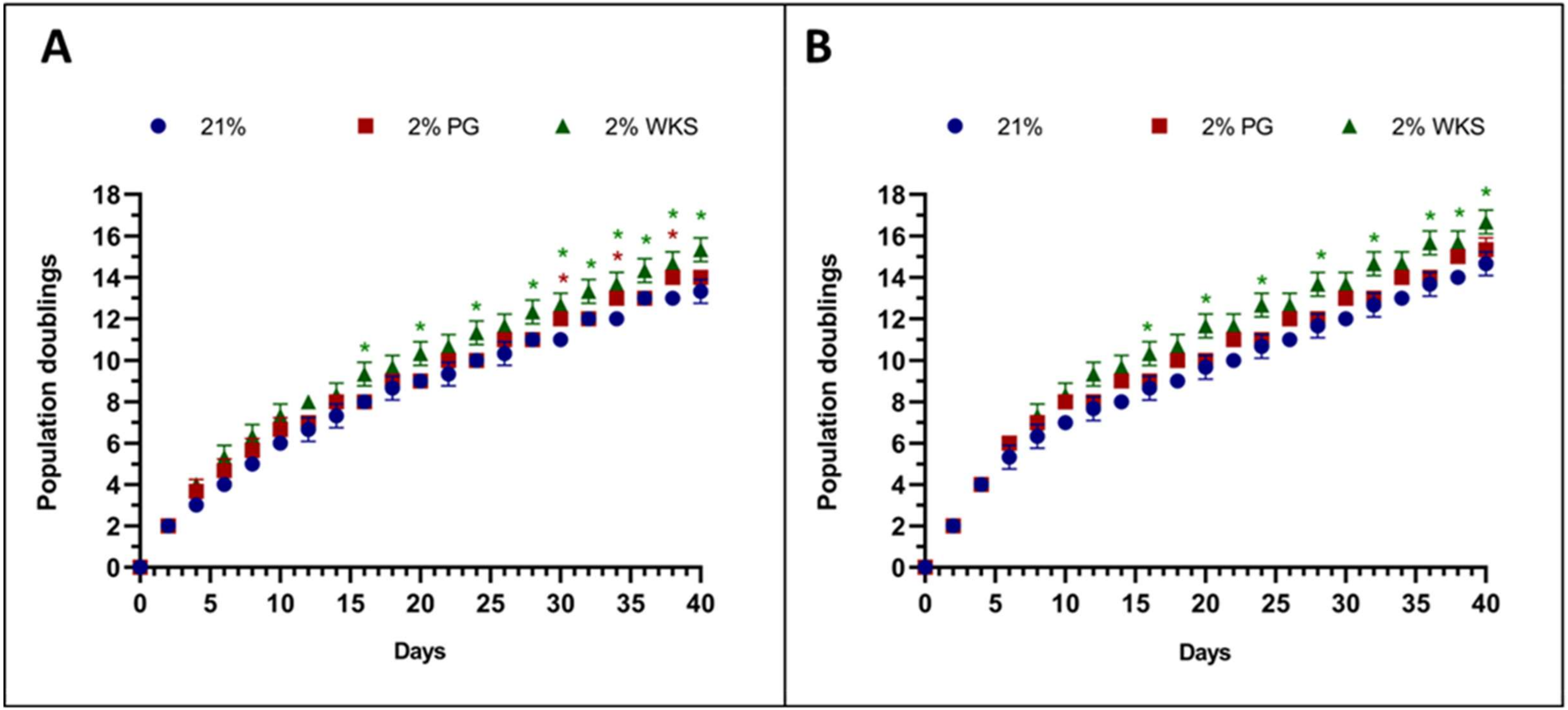

Supplementary Figure S7: Promoter region specific methylation of TERT promoter in SHEF1. Promoter regions (I – V) relative to TSS were evaluated using pyrosequencing in air oxygen (AO) and 2% O2 WKS (WKS). Y-axis indicates DNA methylation (%) at CpG sites, and X-axis indicates sample identity (D indicates day of differentiation) and timepoint of differentiation. Data presented as median (min-max). n=3, *p<0.05, **p<0.01, ***p<0.001.

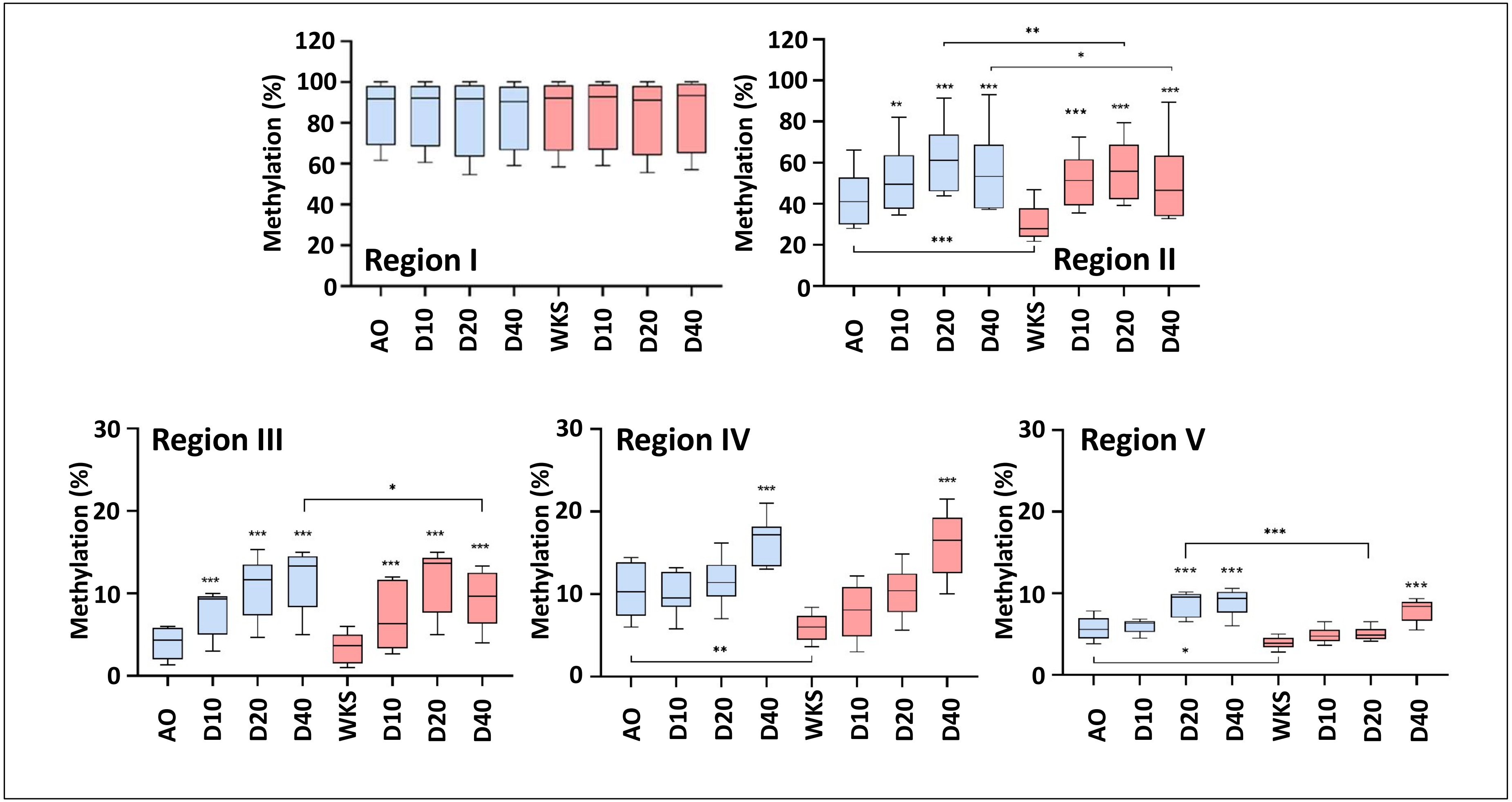

Supplementary Figure S8. Promoter region-specific methylation of TERT promoter in SHEF2. Promoter regions (I – V) relative to TSS were evaluated using pyrosequencing in air oxygen (AO) and 2% O_2 WKS (WKS). Y-axis indicates DNA methylation (%) at CpG sites, and X-axis indicates sample identity (D indicates day of differentiation) and timepoint of differentiation. Data presented as median (min-max). n=3, *p<0.05, **p<0.01, ***p<0.001.

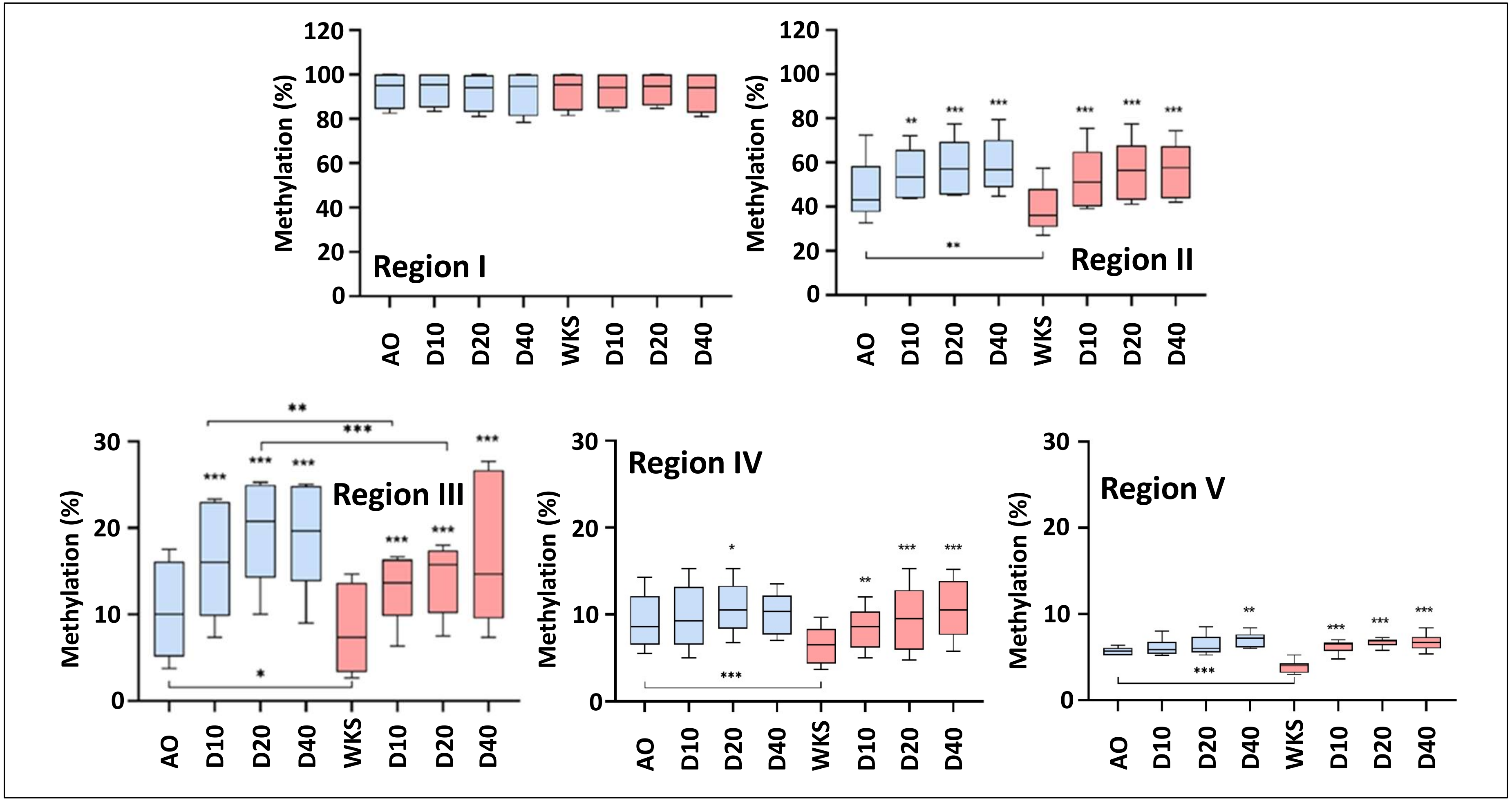

Supplementary Figure S9. DNMT family members display distinct downregulation profiles during hESCs differentiation. DNMT1 (A, B), DNMT3A (C, D) and DNMT3B (E, F) expression data are presented for SHEF1 (A, C, E) and SHEF2 (B, D, F) undifferentiated and differentiated (D) cells in 21% AO and 2% WKS. DNMTs expression (-ΔΔCT) is normalized to the expression of GAPDH. Data are represented as mean ± standard deviation (SD), n=3, *$p<0.05$, **$p<0.01$, ***$p<0.001$ vs 21% AO.

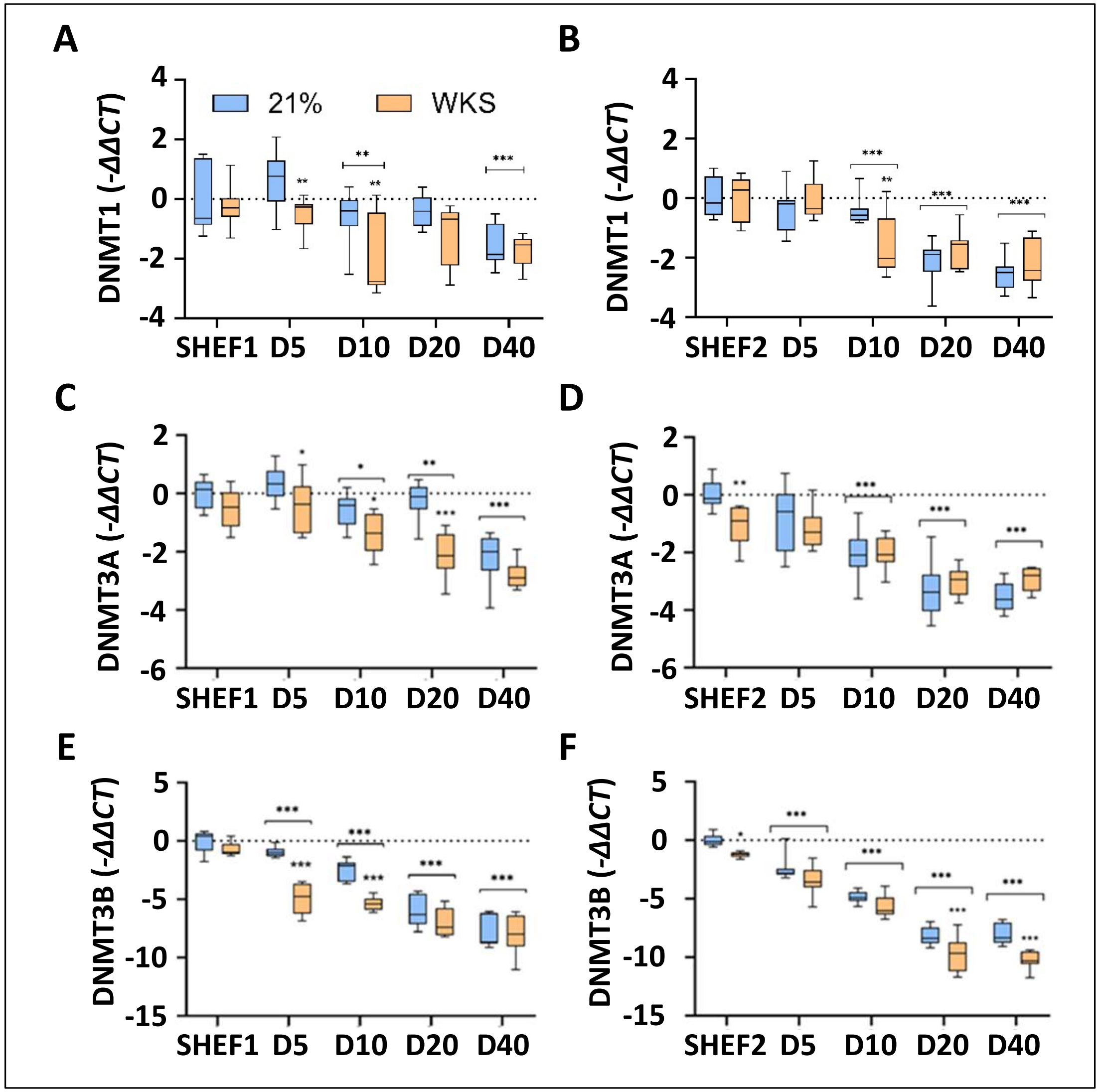

Supplement: Supplementary file 1 [file biomolecules-14-00131-s001.zip › biomolecules-2683122-supplementary.pdf]
